# Steps to Growing Up Healthy: a pediatric primary care based obesity prevention program for young children

**DOI:** 10.1186/1471-2458-14-72

**Published:** 2014-01-23

**Authors:** Amy A Gorin, James Wiley, Christine McCauley Ohannessian, Dominica Hernandez, Autherene Grant, Michelle M Cloutier

**Affiliations:** 1 Department of Psychology, Center for Health, Intervention and Prevention, University of Connecticut, 2006 Hillside Road, Unit 1248, Storrs, CT 06269-1248, USA; 2 Children’s Center for Community Research, Connecticut Children’s Medical Center, 282 Washington Street, Hartford, CT 06106, USA; 3 Children’s Center for Community Research, Connecticut Children’s Medical Center, University of Connecticut Health Center, 282 Washington Street, Hartford, CT 06106, USA

**Keywords:** Weight management, Latino, Low-income, Motivational interviewing

## Abstract

**Background:**

Leading medical organizations have called on primary care pediatricians to take a central role in the prevention of childhood obesity. Weight counseling typically has not been incorporated into routine pediatric practice due to time and training constraints. Brief interventions with simple behavior change messages are needed to reach high-risk children, particularly Latino and Black children who are disproportionately affected by obesity and related comorbidities. Steps to Growing Up Healthy (Added Value) is a randomized controlled trial testing the efficacy of brief motivational counseling (BMC) delivered by primary care clinicians and the added value of supplementing BMC with monthly contact by community health workers (CHW) in the prevention/reversal of obesity in Latino and Black children ages 2-4 years old.

**Methods/Design:**

Mother-child dyads (targeted n = 150) are recruited for this 12-month randomized trial at an inner-city pediatric primary care clinic and randomized to: 1) BMC delivered by clinicians and nurses at well, sick, and WIC visits with the goal of reducing obesogenic behaviors (BMC); 2) BMC plus monthly phone calls by a CHW (BMC + Phone); or 3) BMC plus monthly home visits by a CHW (BMC + Home). During BMC, the medical team facilitates the selection of a specific goal (i.e., reduce sugar sweetened beverage consumption) that is meaningful to the mother and teaches the mother simple behavioral strategies. Monthly contacts with CHWs are designed to identify and overcome barriers to goal progress. Dyads are assessed at baseline and 12 months and the primary outcome is change in the child’s BMI percentile. We hypothesize that BMC + Phone and BMC + Home will produce greater reductions in BMI percentiles than BMC alone and that BMC + Home will produce greater reductions in BMI percentiles than BMC + Phone.

**Discussion:**

Steps to Growing Up Healthy will provide important information about whether a brief primary care-based intervention that utilizes a motivational interviewing and goal setting approach can be incorporated into routine care and is sufficient to prevent/reverse obesity in young children. The study will also explore whether monthly contact with a community health worker bridges the gap between the clinic and the community and is an effective strategy for promoting obesity prevention in high-risk families.

**Trial registration:**

ClinicalTrials.gov NCT01973153

## Background

Populations of color in the United States have disproportionately high rates of childhood obesity [1-3]. Obese children often become obese adults [4,5] setting the foundation for lifelong health disparities. Unfortunately, medical professionals and parents often fail to recognize obesity in young children and parents tend to minimize the risk that obesity poses to their child’s future health outcomes [6-10]. Latina and Black mothers commonly equate excessive weight with good health and interpret a body mass index (BMI) at the 97^th^ percentile or above as a desirable state [11,12]. When families do express interest in weight management, access to treatment is often limited to university-based clinics staffed by highly trained weight management specialists who are able to reach only a handful of children in need. Historically, these weight management programs focus on older children and those who are significantly obese, emphasizing obesity treatment rather than prevention. This model is an important component of obesity management but it is not sufficient. In light of the difficulty and expense of treating obesity once it has developed, widely available cost-effective obesity prevention approaches are needed to promote healthy lifestyles in very young children. Consistent with the Chronic Care Model [13,14], these programs should foster collaborations between health care systems and community resources and form partnerships with families to prevent obesity.

The primary care setting is an obvious context for addressing weight in very young children. Children see their health care provider for regularly scheduled well visits ~10 times before the age of 2 and yearly thereafter [15]. The primary care office thus could provide the continuity of care and frequency of contact needed for weight management in very young children; yet obesity is often not addressed at these visits [16]. O’Brien and colleagues [17] found that among obese children, obesity was documented in only 53% of charts, with diet and physical activity histories reported in only 69% and 15% of charts, respectively. Similar trends were recently reported in a large national sample of pediatricians, with only 55% reporting that they calculated children’s BMIs at well child visits [16]. Consequently, many parents are unaware of their child’s weight status [18] and receive no specific suggestions from their pediatrician regarding how to improve their child’s eating and exercise habits [19,20]. This is particularly true for children under 6 years, those who are overweight but not obese, and African-American, Hispanic, and Asian children [21]. Recognizing this missed opportunity, the American Academy of Pediatrics (AAP) recommends universal assessment of children for obesity risk by their primary care provider [22], a recommendation echoed by the Institute of Medicine’s 2012 report on accelerating progress in obesity prevention [23]. The 2007 AAP obesity guidelines outline a 15-minute obesity prevention protocol to improve early identification of elevated BMI, medical risks and unhealthy habits. These obesity prevention guidelines have not been widely adopted in the primary care setting. In part, this is because the average length of a well child visit is less than 20 minutes [24] and because clinicians often do not feel confident in counseling families about obesity or believe that counseling may not make a difference [20,25,26].

One intervention strategy that shows promise in establishing healthy lifestyles and could fit within the time constraints of primary care is motivational interviewing (MI) [27,28]. Using patient-centered strategies such as open-ended questions, positive affirmations and reflective listing, MI elicits internal motivation for behavior change while addressing the ambivalence and discrepancies between a person’s current values and behavior (e.g., “Heavy is healthy”) and their future goals (e.g., “I don’t want my child to get diabetes.”). This approach is ideal for primary care provider use because MI can be delivered in brief doses. Research has shown MI to be effective across a range of health behaviors, including diet and nutrition-focused interventions [29-31]. MI has been used successfully to supplement behaviorally-based adult weight loss programs and has been shown to have a positive impact after only 2 sessions [30]. In pediatric settings, MI has been associated with increased parent satisfaction and adherence and has been used in a nonrandomized childhood obesity prevention program [32]. More research is needed to test whether brief but recurrent doses of MI coupled with key behavioral strategies known to promote weight control (e.g., goal setting and self-monitoring) can be integrated into primary care to prevent childhood obesity and to explore how best to support the behavior change process outside of the clinic setting.

Community health workers (CHWs) bridge the gap between the primary care office and the community and may be a viable resource for reinforcing obesity prevention efforts once families leave the doctor’s office. CHWs are typically individuals from within a community who share many demographic similarities and life experiences with their target audience [33]. As a trusted member of a community, CHWs are able to offer culturally appropriate health education, counseling, and social support that can facilitate access to information and resources [34]. CHWs are being used extensively to address disease and case management, health information transfer, and health promotion and have been effective in promoting behavior change in multiple settings [35]. CHWs have had a positive influence on diabetes self-management [36] and breastfeeding outcomes [37] as well as on general nutrition knowledge and dietary intake behaviors among Latinos. A question that remains unanswered is whether CHWs can enhance obesity prevention outcomes above and beyond what can be achieved through primary care.

The present study is a randomized controlled trial designed to examine the efficacy of Steps to Growing Up Healthy, a primary care based obesity prevention program that utilizes a motivational interviewing framework and selected behavioral strategies to reduce obesogenic behaviors in Latino and Black children 2-4 years of age. The behavioral targets are reducing/eliminating sugar sweetened beverage consumption, changing the type and/or amount of milk consumed, decreasing screen time to less than 2 hours per day, and increasing physical activity to at least 60 minutes per day. The primary goal of this study is to examine whether repeated doses of brief motivational counseling delivered by primary care clinicians and nurses to mothers of young children are sufficient to prevent/reverse childhood obesity in this high risk group or if monthly contacts with a CHW via either telephone or home visits enhances any observed intervention effects. This project is innovative in that the initial obesity prevention activities are embedded in the context of routine clinic visits and are tested in combination with two modalities for providing CHW support. The study’s focus on preschool age children will also add to the small but growing literature on obesity prevention in this critical developmental window.

## Methods/Design

### Overview and hypotheses

**Figure 1:**
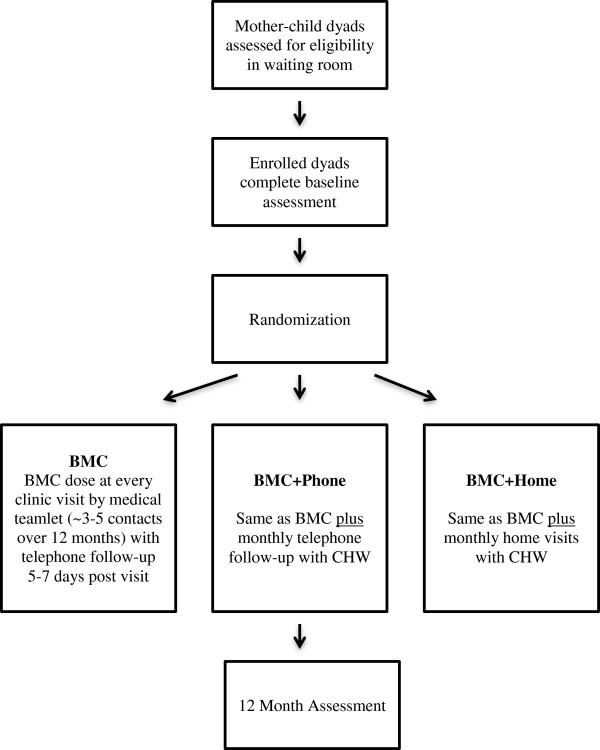
Study flow.

Latino and Black mother-child dyads (targeted *n* = 150) are recruited for this 12-month randomized trial at an urban based pediatric primary care clinic (Figure 1). Participating dyads are assigned with equal probability to one of three treatment conditions: 1) brief motivational counseling (BMC) alone delivered by the child’s medical team; 2) BMC plus monthly phone calls by a CHW (BMC + Phone); or 3) BMC plus monthly home visits by a CHW (BMC + Home). Dyads are assessed at baseline and 12 months. The primary outcome is change in the child’s BMI percentile. We hypothesize that over the 12-month period both BMC + Phone and BMC + Home will produce greater reductions in BMI percentiles than BMC and that BMC + Home will produce greater reductions in BMI percentiles than BMC + Phone. The study protocol was reviewed by the Scientific Review Committee at Connecticut Children’s Medical Center (CCMC) in Hartford, CT and received full approval by CCMC’s Institutional Review Board.

### Participants

Mother-child dyads are recruited from the Primary Care Clinic (PCC) at Connecticut Children’s Medical Center. Mothers of 2-4 year olds have been targeted because of the frequent contact this age group has with their primary care clinician and because our recent data [38] demonstrates that obesogenic habits are established early, often by the preschool years. To participate, the child must be between 2-4 years old, of Latino or Black descent by maternal report, and be receiving services through the Special Supplemental Nutrition Program for Women, Infants, and Children (WIC) to ensure frequent clinical contact at the PCC over the course of 12 months. There is no BMI cutoff as even children of normal weight with obesogenic behaviors may benefit from the intervention. If there is more than one eligible child in a family, data from the first child enrolled in the study will be used for analysis. Dyads are excluded from participating if the mother is younger than 18 years old, if the dyad does not live in the Greater Hartford area or if they have plans to move out of the area in the next 12 months, or if the child or mother has special needs (dietary, physical, and/or emotional) that would make the intervention inappropriate (e.g., failure to thrive, type 1 diabetes, cystic fibrosis).

### Recruitment and randomization

Mothers attending the clinic with their child for a well child or WIC visit are approached in the waiting room by research staff who describe the study and obtain informed consent from mothers who are interested and meet eligibility criteria. Research staff then administers the baseline assessment battery and randomize the dyads into one of the three treatment conditions using a block randomization process. Dyads receive their first dose of the intervention at this clinic visit.

### Interventions

#### Common intervention components across all three conditions

##### Overview

The obesity prevention approach we are testing starts with pediatric primary care clinicians and strives to form a partnership between a mother and her child’s medical team using brief motivational interviewing and selected behavioral strategies (BMC). Mother-child dyads randomized to the added value conditions (BMC + Phone and BMC + Home) build upon that relationship using CHWs similarly trained in brief motivational interviewing who reinforce the behavior change messages and provide families with additional support and assistance in reaching their selected goals. The intervention has been designed with extensive feedback from families, the medical team, and the CHWs. A practice champion at the primary care clinic with a longstanding interest in childhood obesity is serving as a liaison between the research team and clinicians to facilitate the implementation of BMC at the clinic.

##### Behavioral targets

The intervention centers on 4 key behavioral targets: reduce/eliminate sugar sweetened beverage consumption, change the type and/or quantity of milk consumed, decrease screen time to less than 2 hours per day, and increase physical activity to at least 60 minutes per day. While there are many potential behaviors to target to prevent childhood obesity, we highlight these four because there are clear age-appropriate guidelines about these behaviors from the AAP [22] and because focus groups with mothers demonstrated their interest and willingness to implement changes in these areas. There is flexibility in goal setting, however, and mothers are encouraged to select a goal that is personally meaningful even if it is outside of these key behaviors.

##### Toolkit

At study enrollment, all dyads receive a study toolkit that contains low-cost items to assist in their behavior change efforts. Toolkit items include a 6 ounce spill-proof cup, a measuring cup labeled with appropriate serving sizes for milk and sugar sweetened beverages, a placemat with examples of portion sizes for young children, a foam ball to encourage physical activity, and a pedometer for mothers.

##### BMC

**Figure 2:**
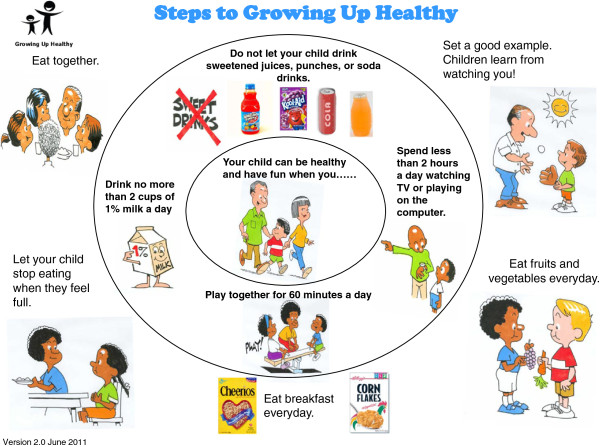
Steps to growing up healthy behavior target clinical tool.

All dyads in the study receive BMC delivered by clinicians and nurses at routine medical visits (well, sick, or WIC check-in) over a 12-month period. As part of standard care, every child in the primary care clinic is assigned to one of two teams consisting of attending clinicians, pediatric residents, nurses and medical assistants. Each child on the team is assigned to a “teamlet” (an attending clinician and nurse) who provides all of that child’s care [39,40]. Well child visits and the WIC visits are performed by the child’s teamlet while sick visits may be conducted by others on the child’s team. Any trained clinician or nurse seeing the family for any type of visit can offer the intervention. Each BMC dose is approximately 3-5 minutes long and incorporates basic MI strategies such as positive affirmations and reflective listening as well as behavioral elements including goal setting and contracting. Seven intervention steps guide this brief encounter (Table 1). First, in the waiting room, mothers complete a one-page survey created for this project (Steps to Growing Up Healthy Survey) regarding their child’s eating, physical activity, sedentary activity, and sleep habits (Step 1). At the start of BMC, the medical teamlet member quickly reviews the survey results with the family focusing first on areas of strength using a colorful circular diagram in English and Spanish (Step 2; Figure 2). The clinician then selectively focuses the mother’s attention on the four key behavioral targets that form the inner circle of the Steps to Growing Up Healthy circular diagram (Step 3). Using open-ended questions and reflective listening skills, the clinician and mother agree upon a behavior that the mother is ready and able to change (Step 4) and a plan of action specific for that child is agreed upon and documented in a written behavioral contract that clearly states the mother’s goal (Step 5; i.e., the Steps to Growing Up Healthy Action Plan). The clinician or nurse then offers the mother an educational handout containing suggestions for how to implement the desired behavior change (Step 6). The BMC dose ends with the clinician or nurse providing the mother with a monthly self-monitoring calendar that the mother can use to track goal progress via a simple yes/no daily checkbox (Step 7). At every visit during the next 12 months, the clinician and nurse are encouraged to use the Steps to Growing Up Healthy Survey and BMC with targeted communication to reinforce a previously agreed upon goal or to deliver a new behavior change message and set a new goal. In addition, within 5-7 days of each clinic visit, a member of the project staff conducts a brief (≤5 minutes) telephone call with the mother to review the visit, discuss initial implementation of behavior change and any problems encountered with the behavior change, and assess the fidelity of the intervention rendered by the medical teamlet according to the mother’s perception of the interaction.

**Table 1:** Intervention elements included in a brief motivational counseling (BMC) dose delivered in the primary care setting

**Step**	**Intervention activity**	**Approximate time**
Step 1	Mother completes Steps to Growing Up Healthy Survey in the waiting room providing information on child’s eating, activity, and health habits	5 minutes
Step 2	Clinician/nurse reviews Steps Survey with mother, affirms areas of positive health behaviors	1 minute
Step 3	Clinician/nurse focuses mother’s attention on 4 key behavioral targets, assesses interest and confidence in addressing areas where mother reports obesogenic behaviors	1 minute
Step 4	Clinician/nurse and mother agree upon behavior the mother is ready and able to change	30 seconds
Step 5	Behavioral goal is document using a written contract signed by clinician/nurse and mother	30 seconds
Step 6	Clinician/nurse provides mother with an educational handout specific to the selected goal	30 seconds
Step 7	Clinician/nurse provides monthly self-monitoring calendar for mother to track goal progress	30 seconds

To encourage medical teamlets to use BMC with their patients, clinicians and nurses receive $10 each time they provide a BMC dose. The clinician/nurse must document the child’s BMI percentile, discuss an obesogenic behavior from the Steps to Growing Up Healthy Survey using BMC, and create a Steps to Growing Up Healthy Action Plan in concert with the family to receive the incentive. Over the course of the 12-month intervention period, we anticipate that families will receive 3-5 doses of BMC in the clinic (1-2 well child visits, 1-2 sick visits, and 1-2 WIC check-ins) and 3-5 additional telephone follow-up calls.

#### Treatment components specific to the added value conditions

##### BMC + Phone

Mother-child dyads randomized to BMC + Phone receive BMC doses at clinic visits as described above plus every month a CHW calls the mother to assess how well the family is doing with their selected behavior change, discuss any barriers the family is experiencing in implementing the behavior change, review the monthly self-monitoring calendar, reinforce positive behaviors, assess the mother’s confidence in achieving her goal, and assist the mother to implement her goal through progressive steps and gradual implementation of new behaviors. Behavioral goals are modified if mothers report having met their goals and/or express interest in selecting a new goal. Each phone call is approximately 10-15 minutes long.

##### BMC + Home

Mother-child dyads randomized to BMC + Home receive BMC doses at clinic visits as described above plus monthly home visits by a CHW. The focus of these hour long home visits is similar in content to BMC + Phone (i.e., CHWs assess behavior change progress, discuss barriers, review self-monitoring, assist the mother in implementing her goal and/or behavior change by breaking it into small, manageable steps, assess the mother’s confidence in making the behavior change). In addition, the CHW assists the mother with label reading and meal planning, how to structure her home environment to support healthy diet and activity choices, how to determine appropriate portion sizes, and how to enhance physical activity. Activities are visual, interactive, and based on a goal setting and motivational interviewing approach.

#### Interventionist training

##### Primary care staff

All clinicians and nurses at the PCC (*n* = 32) were invited to participate in the study and receive training in motivational interviewing provided by the study investigators and an external consultant from the Motivational Interviewing Network of Trainers. Clinicians and nurses were asked to attend two 2-hour sessions focusing on key MI strategies such as asking open-ended questions, using reflective listening, positive affirmation, decreasing resistance, and assessing the mother’s interest and confidence in making change. A treatment manual was provided that included a detailed outline of the BMC intervention steps. Mini-booster sessions were conducted periodically at staff meetings and with individual clinicians throughout the intervention period to encourage on-going use of BMC and to assure intervention fidelity. Twenty-four clinicians and nurses provided consent allowing us to collect information about their attitudes towards obesity management and comfort with MI.

##### Community health workers

Two certified CHWs from AHEC (Central Area Health Education Center, Inc.) were hired and received a total of 84 hours of training. Forty eight hours of training were completed by AHEC and focused on core competencies in the CHW. Thirty-six hours of additional training were conducted in motivational interviewing and in project implementation. Approximately 1/3 of this training included intervention modification to make the materials more interactive and culturally relevant. A CHW manual was developed by adapting existing resources developed by WIC, the Cooperative Extension System, and the Parents as Teachers model that was recently demonstrated to be effective in changing pre-school eating habits [41] to the health literacy and cultural needs of our target community.

### Outcome measures

The following measures are administered at baseline and 12 months unless otherwise noted.

#### Child measures

##### Height and weight

The primary outcome is change in the child’s BMI percentile. Height is measured with the child barefoot and erect against a wall-mounted stadiometer and recorded to the last 0.5 cm. Children are weighed in light clothing to the last complete 100 g. BMI percentile is calculated using age and sex-specific information from the 2000 revised CDC/NCH growth charts for the United States [42].

##### Diet and physical activity

We are using the parent-report 24-item Children’s Dietary Questionnaire to assess dietary quality [43]. This measure is appropriate for preschool age children and all subscales have demonstrated satisfactory test-retest reliability and an ability to detect change in the expected direction following a weight management intervention. To assess physical and sedentary activity, we are using a brief 12-item questionnaire by Spurrier et al. [44] based on the Outdoor Playtime Checklist. The questionnaire assesses outdoor playtime and has been validated against objective accelerometer data. It also assesses sedentary activity focusing on small screen entertainment (i.e., time spent watching TV and playing video games). This measure has been used in prior studies of preschool aged children and is sensitive to change over time. In addition, the Steps to Growing Up Healthy Survey, a 16-item questionnaire developed specifically for this study is administered at every clinic visit to assess the 4 target areas as well as the child’s general health habits (e.g., water consumption, sleep).

#### Maternal measures

##### Demographics

Basic demographic information (e.g., gender, age, primary language, household living situation, employment status, household income) is obtained via self-report from mothers.

##### Acculturation (Baseline only)

We ask Hispanic mothers to complete the Brief Acculturation Rating Scale for Mexican Americans II [45]. This 12-item scale measures acculturation along three factors (language, ethnic identity, and ethnic interaction) and has been used with Mexican Americans and individuals of Puerto Rican decent [45,46].

##### Feeding practices

Mothers complete the 19-item Caregiver’s Feeding Styles Questionnaire, a well validated and frequently used measure of the demandingness and responsiveness of parental feeding practices [47-49].

##### Additional maternal variables

We are assessing 5 potential moderators that might influence treatment response including a 15-item food insecurity questionnaire [50], the 4-item Perceived Stress Scale [51], a 2-item maternal depression screening tool [52], the 15-question Health Care Climate Questionnaire [53] that assesses the mother’s perceptions of the degree to which her child’s medical team provides autonomy support, and an abbreviated version of the Neighborhood Environment Walkability Scale [54,55].

##### Process evaluation (12 months only)

At the end of the intervention period, we ask mothers to evaluate the program they received, whether they found it helpful, which components were most useful, and whether they would refer a friend to receive the same program.

#### Medical teamlet measures

After obtaining consent and prior to BMC training, clinicians, and nurses completed a set of questionnaires that address self-efficacy, outcome value and outcome expectancy related to obesity prevention in children, clinician-parent interactions around obesity, as well as their experience and thoughts regarding BMC and knowledge regarding obesity.

##### Demographic survey

Job status (full time vs. part time), tenure (length of time in the PCC), number of years since receiving highest degree, and Spanish fluency are assessed via self-report.

##### The office weight management survey

This 52-question instrument assessed clinical goals for obesity prevention/reversal, time spent in obesity-related activities, self-efficacy, outcome value and expectancy and barriers to obesity prevention/reversal. The instrument was made specific for obesity from a general pediatric instrument [56] and from the recommendations of the AAP’s Obesity Task Force.

##### Healthy living questionnaire

This 20-item questionnaire examines clinician knowledge surrounding healthy behaviors for two year olds and is based upon the 2007 AAP Guidelines.

##### Hope and conscientiousness

The personality traits of hope and conscientiousness have been found in previous research to predict goal directed behavior by primary care clinicians [57]. We assess these traits among clinicians and nurses at baseline using the 4 agency and 4 pathway statements of the Hope Scale [58] and the Five Factor Inventory [59] in addition to a 10-item obstacles scale specifically related to work obstacles in a primary care setting [57].

#### Adherence and treatment fidelity

We are recording the number of BMC visits completed in total and by each member of the medical teamlet (i.e., number of BMC doses delivered; number of signed action plans) and by CHWs, as well as the number of telephone contacts completed by CHWs and study staff.

On each post-clinic visit phone call that is completed within a week of each BMC dose, study staff are assessing mothers’ perceptions of their clinic encounter. Specifically, mothers are asked to respond no, somewhat, or yes to whether the doctor or nurse 1) reviewed their responses to the Steps to Growing Up Healthy Survey, 2) told them some things they were doing right in terms of their child’s eating and exercise habits, 3) talked to them about areas in which they could make changes to improve their child’s nutrition and physical activity, and 4) asked in what specific area they would like to make changes in. Mothers are also asked if the doctor or nurse selected the behavioral goal or if this was decided upon together.

### Retention

Proactive efforts are made to retain families for the 12-month study period. To aid in locating families for follow-up assessments, contact information (name, address, and phone number) of a family member and friend is obtained at the time of enrollment and at the time of each visit. As part of the Steps to Growing Up Health study, families are given an honorarium of $25 at baseline and at 12 months for completing these assessments visits (for a possible total of $50). Families in BMC + Phone and BMC + Home receive $5 per telephone call or home visit for a possible additional total of $60.

### Sample size and power

The primary outcome is change in child’s BMI percentile from baseline to 12 months. We hypothesize that both BMC + Phone and BMC + Home will reduce BMI percentiles compared to BMC. Assuming no change in BMI percentile for BMC and a 2.5 percentile change in BMI for BMC + Phone (i.e., 90^th%^ to 87.5^th%^) and a 5 percentile change in BMI in BMC + Home (i.e., 90^th%^ to 85^th%^), with an alpha of 0.05 and an ANCOVA with 3 treatment groups, we need 34 children per group to have 0.85 power to be able to perform 3 contrasts: BMC vs. BMC + Phone, BMC vs. BMC + Home, and BMC + Phone vs. BMC + Home. We plan to recruit 50 children per arm to allow for a 15% dropout rate. Even if 25% of participants drop out we will have 80% power to test our primary aims.

### Statistical analysis

#### Primary analysis

We will use an-intent-to-treat approach to data analysis. All randomized children will be included in the analyses regardless of whether they receive any actual intervention. We will impute BMI percentile change among attritors using their historical heights and weights obtained through chart review; however, we also will conduct treatment completer analyses using only complete cases. Baseline variables (e.g., demographics) will be compared between intervention groups using chi-square statistics and *t*-tests to determine if any group differences exist despite randomization. Baseline variables that differ between groups will be included as covariates in subsequent analyses.

Repeated measures analysis of variance models will be conducted to address the research questions. In the primary analysis, the dependent variable will be BMI percentile. In this model, the within-subject factor will be time, which will include 2 levels (baseline and 12 months). Between-subjects factors will include treatment group (BMC, BMC + Phone, and BMC + Home). Demographic variables that are found to differ across groups will be included as covariates.

#### Secondary analyses

A similar repeated measures ANOVA approach will be used to examine intervention effects on obesogenic behaviors. The within-subjects and between-subjects factors will be identical to those in the primary model above and the dependent variables will be diet and physical activity. We will also examine dose-response effects of the interventions using regression models predicting 12 month BMI percentile from the number of doses of intervention received controlling for baseline BMI percentile. Linear mixed modeling will be used to determine if maternal (depression, perceived stress, health care climate) and family (food insecurity, acculturation) characteristics have main effects on change in BMI percentiles and, of greater interest, if they moderate treatment group effects. In this analysis, BMI percentiles at baseline and 12 months will be used as a repeated variable, intervention group will be used as a fixed subject variable, and maternal and family characteristics will be covariates (first individually and then jointly) to examine how they relate to changes in weight across the sample. Then, interactions between intervention group and maternal and family characteristics will be entered into the model to determine if any maternal/family characteristics moderate the effect of intervention group on changes in BMI percentiles. Multiple regression analyses will be used to determine if clinician’s attitudes towards obesity, BMC self-efficacy, hope and conscientiousness and outcome expectancy predict the number of doses of BMC that they deliver. Finally, as Steps to Growing Up Healthy represents an example of a “practical trial” we plan to evaluate the RE-AIM criteria (Reach; Effectiveness; Adoption; Implementation; Maintenance) [60] by looking at indicators such as the percentage of mothers approached about the study who enroll, the percentage of mother-child dyads who receive more than one dose of BMC, and the number of signed action plans.

## Discussion

Obesity is recognized by pediatric primary care clinicians as the most significant health problem facing families today [61] yet it is often not addressed as part of routine care. Leading medical organizations including the American Academy of Pediatrics and the Institute of Medicine have called on primary care providers to expand their role in obesity prevention; however, time constraints, lack of training in obesity management, and concerns about the minimal impact of weight counseling contribute to the limited implementation of current prevention guidelines. For progress to be made in this area, simple diet and exercise action plans are needed that can be incorporated into pediatricians’ busy ongoing practices without extensive training requirements on the part of clinicians [16].

Steps to Growing Up Healthy is a randomized controlled trial testing an evidence-based intervention for mothers of Latino and Black children 2-4 years of age that offers repeated doses of brief motivational interviewing coupled with core behavioral strategies (i.e., goal setting, self-monitoring) targeting four areas of behavior change (i.e., sugar sweetened beverage consumption, milk consumption, screen time, physical activity). The doses are administered by the child’s existing medical team (the medical teamlet comprised of a primary care clinician and a nurse) during routine visits over the course of one year. We are examining the efficacy of this approach compared to the added value of offering monthly telephone follow-up or home visits by community health workers. We designed the core intervention to fit within regularly scheduled primary care visits to reduce the burden on the medical team and families with the expectation that a dose will be provided at every clinic visit. Guided by the Chronic Care Model, we are utilizing bilingual/bicultural CHWs who have children of their own and reside in the target community to facilitate adoption and maintenance of weight-related behavior change by problem-solving with families about barriers to successful goal progress and reinforcing behavior change messages initially received in the primary care office. The study is innovative in its focus on very young children, the use of routine clinic visits to address obesity management, and the testing of two different types of contact with CHWs in the prevention/reversal of obesity.

## Competing interests

The authors declare that they have no competing interests.

## Authors’ contributions

AAG and MMC designed, planned, and oversaw all scientific aspects of the study. JW made contributions to the study design and oversaw acquisition of data. DH participated in the training of interventionists and in the acquisition of data. AG was responsible for database management. CO was involved in the drafting of the manuscript and devising the analytic plan. All authors read and approved the final manuscript.

## Pre-publication history

The pre-publication history for this paper can be accessed here:

http://www.biomedcentral.com/1471-2458/14/72/prepub
